# Proteomic Characterization of Acute Kidney Injury in Patients Hospitalized with SARS-CoV2 Infection

**DOI:** 10.21203/rs.3.rs-2379226/v1

**Published:** 2023-03-16

**Authors:** Girish Nadkami, Ishan Paranjpe, Pushkala Jayaraman, Chen-Yang Su, Sirui Zhou, Steven Chen, Diane Del Valle, Ryan Thompson, Ephraim Kenigsberg, Shan Zhao, Suraj Jaladanki, Kumardeep Chaudhary, Steven Ascolillo, Akhil Vaid, Edgar Gonzalez-Kozlova, Arvind Kumar, Manish Paranjpe, Ross O’Hagan, Samir Kamat, Faris Gulamali, Justin Kauffman, Hui Xie, Joceyln Harris, Manishkumar Patel, Kimberly Argueta, Craig Batchelor, Kai Nie, Sergio Dellepiane, Leisha Scott, Matthew Levin, John He, Mayte Suárez-Fariñas, Steven Coca, Lili Chan, Evren Azeloglu, Eric Schadt, Noam Beckmann, Sacha Gnjatic, Miriam Merad, Seunghee Kim-Schulze, J. Brent Richards, Benjamin Glicksberg, Alexander Charney

**Affiliations:** Icahn School of Medicine at Mount Sinai; Hasso Plattner Institute for Digital Health at Mount Sinai, Icahn School of Medicine at Mount Sinai, New York, NY USA; Icahn School of Medicine at Mount Sinai; McGill University; Lady Davis Institute; Icahn School of Medicine at Mount Sinai; Icahn School of Medicine at Mount Sinai; Icahn School of Medicine at Mount Sinai; Icahn School of Medicine at Mount Sinai; Icahn School of Medicine at Mount Sinai; Icahn School of Medicine at Mount Sinai; Icahn School of Medicine at Mount Sinai; Icahn School of Medicine at Mount Sinai; Icahn School of Medicine at Mount Sinai; Mt Sinai School of Medicine; Icahn School of Medicine at Mount Sinai; Harvard Medical School; Icahn School of Medicine at Mount Sinai; Icahn School of Medicine at Mount Sinai; Icahn School of Medicine at Mount Sinai; Icahn School of Medicine at Mount Sinai; Icahn School of Medicine at Mount Sinai; Icahn School of Medicine at Mount Sinai; Icahn School of Medicine at Mount Sinai; Icahn School of Medicine at Mount Sinai; Icahn School of Medicine at Mount Sinai; Icahn School of Medicine at Mount Sinai; Icahn School of Medicine at Mount Sinai; Icahn School of Medicine at Mount Sinai; Icahn School of Medicine at Mount Sinai; Mount Sinai School of Medicine; Icahn School of Medicine at Mount Sinai; Icahn School of Medicine at Mount Sinai; Icahn School of Medicine at Mount Sinai; Icahn School of Medicine at Mount Sinai; Icahn School of Medicine at Mount Sinai; Icahn School of Medicine at Mount Sinai; Icahn School of Medicine at Mount Sinai; Icahn School of Medicine at Mt. Sinai; Lady Davis Institute for Medical Research, Jewish General Hospital; Icahn School of Medicine at Mount Sinai; Icahn School of Medicine at Mount Sinai

## Abstract

**Background:**

Acute kidney injury (AKI) is a known complication of COVID-19 and is associated with an increased risk of in-hospital mortality. Unbiased proteomics using biological specimens can lead to improved risk stratification and discover pathophysiological mechanisms.

**Methods:**

Using measurements of ~4000 plasma proteins in two cohorts of patients hospitalized with COVID-19, we discovered and validated markers of COVID-associated AKI (stage 2 or 3) and long-term kidney dysfunction. In the discovery cohort (N= 437), we identified 413 higher plasma abundances of protein targets and 40 lower plasma abundances of protein targets associated with COVID-AKI (adjusted p <0.05). Of these, 62 proteins were validated in an external cohort (p <0.05, N =261).

**Results:**

We demonstrate that COVID-AKI is associated with increased markers of tubular injury (*NGAL*) and myocardial injury. Using estimated glomerular filtration (eGFR) measurements taken after discharge, we also find that 25 of the 62 AKI-associated proteins are significantly associated with decreased post-discharge eGFR (adjusted p <0.05). Proteins most strongly associated with decreased post-discharge eGFR included *desmocollin-2, trefoil factor 3, transmembrane emp24 domain-containing protein 10*, and *cystatin-C*indicating tubular dysfunction and injury.

**Conclusions:**

Using clinical and proteomic data, our results suggest that while both acute and long-term COVID-associated kidney dysfunction are associated with markers of tubular dysfunction, AKI is driven by a largely multifactorial process involving hemodynamic instability and myocardial damage.

## Introduction

Severe acute respiratory syndrome coronavirus 2 (SARS-CoV-2) is a novel coronavirus that has caused the coronavirus disease 2019 (COVID-19) pandemic. Although effective vaccines are available, novel variants that may evade neutralizing antibodies exist in the population and have led to high case counts and periodic case surges. COVID-19 most commonly presents with fever, cough, and dyspnea^1,2^ and is associated with acute respiratory distress syndrome (ARDS). However, the clinical syndrome resulting from SARS-CoV-2 infection is broad, ranging from asymptomatic infection to severe disease with extrapulmonary manifestations^3^, including acute kidney injury^4^, acute myocardial injury^5,6^ and thrombotic complications^7–11^. The CRIT-COV-U research group in Germany recently developed a urinary proteomics panel COV50 that could consider this variability in infection by generating biomarkers that can indicate adverse COVID-19 outcomes based on the WHO severity scale^12^

Acute kidney injury (AKI) is a particularly prominent complication. The rates of AKI vary greatly based on patient population, but evidence suggests that at least 30% of hospitalized patients and 50% of patients in the intensive care unit (ICU) develop AKI^1,4,13–16^. Although the rate of AKI in hospitalized COVID-19 patients has decreased since the initial surge in 2020, the incidence remains high^17^. Like community-acquired pneumonia^18^, AKI is increasingly recognized as a common complication of COVID-19 in the hospitalized setting and confers significantly increased morbidity and mortality^19^.

There is a limited understanding of the pathophysiology of COVID-19-associated AKI. A recent paper^20^ compared transcriptomics and proteomics of postmortem kidney samples of patients with severe COVID-19 and autopsy-derived control cohorts of sepsis-AKI and non-sepsis-AKI. The work found common inflammatory pathways and regulatory responses including the downregulation of oxidative signaling pathways between COVID-19 AKI and sepsis-AKI. They also confirmed the observation of tubular injury in almost all their COVID-19 AKI samples while drawing similarities between the inflammation response of sepsis-associated AKI and COVID-19 associated AKI. Histopathological reports from autopsy specimens have provided conflicting insights into the pathological changes in the kidney in COVID-19. A report of 26 patients who died with COVID-19 AKI revealed acute tubular injury as a prominent mechanism^21^. Additionally, the presence of viral particles in the tubular epithelium and podocytes in autopsy specimens has been reported^21,22^, which is evidence of direct viral invasion of the kidney. In addition, coagulopathy and endothelial dysfunction are hallmarks of COVID-19^23^ and may also contribute to AKI. Finally, SARS-CoV-2 may directly activate the complement system^24^. In addition to these mechanisms, systemic effects of critical illness (hypovolemia, mechanical ventilation) and derangements in cardiac function and volume may also contribute to COVID-19 AKI.

In addition to morbidity and mortality in the acute setting, COVID-19 is also associated with long term manifestations i.e., the post-acute sequelae of SARS-CoV2 (PASC)^25^. Kidney function decline is a major component of PASC and a study of more than 1 million individuals found that survivors of COVID-19 had an elevated risk of post-acute eGFR decline^26^, suggesting long term kidney dysfunction may occur following the acute infection.

Given the high incidence of COVID-19 associated kidney dysfunction, the unknown pathophysiology, and the urgent need for better approaches for risk stratification for long term kidney function decline we aimed to characterize the proteomic changes associated with COVID associated AKI and long-term kidney function. Proteomic biomarkers have previously shown success in predicting COVID-19 outcomes^27–29^. Other work^12^ has also applied urinary proteomic profiling to predict worsening of COVID-19 at early stages of the infection. Prior research using minimally invasive proteomics assays supports the use of peripheral serum as a readily accessible source of proteins that accurately reflect the human disease state^30–33^. We measured protein expression of more than 4000 proteins from serum samples collected in a diverse large cohort of hospitalized patients with COVID-19 and validated significant results in an independent cohort and identified proteins that are significantly different between patients with and without AKI. We then determined whether these proteomic perturbations also characterize post-discharge kidney function decline as measured by estimated glomerular filtration rate (eGFR).

## Materials And Methods

### Patient cohort

**Figure 1:**
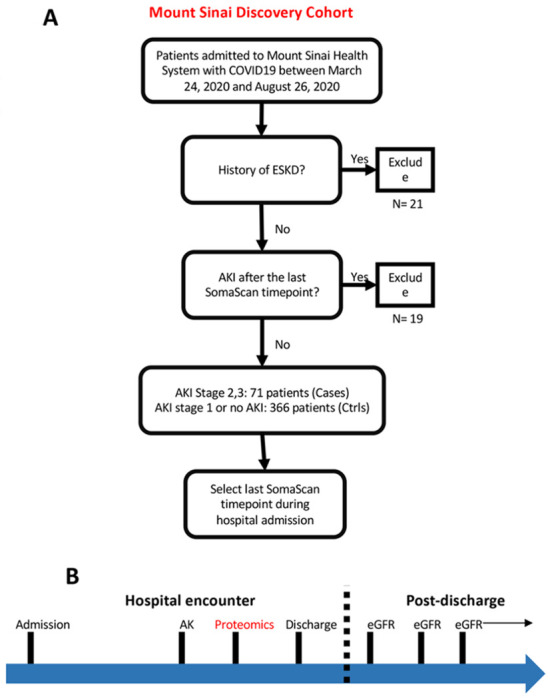
Overview of the discovery cohort selection process. **a.** Cohort selection strategy overview, **b.** eGFR measurements recorded post-discharge for returning patients until 12/21/2021. **A**: Prospective cohort of patients enrolled between March 2020 – Aug 2020 **B**: Timeline of measurements taken

An overview of the discovery cohort selection process is provided in Fig 1. We prospectively enrolled patients hospitalized with COVID-19 between March 24, and August 26, 2020, at five hospitals of a large urban, academic hospital system in New York City, NY into a cohort as previously described^34^. The cohort enrolled patients who were admitted to the health care system with a COVID-19 infection and had broad inclusion criteria without specific exclusion criteria. The Mount Sinai Institutional Review Board approved this study under a regulatory approval allowing for access to patient level data and biospecimen collection^35^. Peripheral blood specimens were collected at various points during the hospital admission for each patient.

The validation cohort included a prospective biobank from Quebec, Canada that enrolled patients hospitalized with COVID-19, as previously described^29^. Patients were recruited from the Jewish General Hospital and Centre Hospitalier de l’niversité de Montréal. Peripheral blood specimens were collected at multiple time points after admission.

We defined an AKI cohort using proteomic data acquired at the last available timepoint during the hospital course for all individuals. Patients who developed AKI after the last specimen collection timepoint were excluded. Controls were defined as individuals who developed AKI stage 1 or did not develop AKI during their hospital course.

### Serum collection and Processing

Blood samples were collected in Serum Separation Tubes (SST) with a polymer gel for serum separation as previously described^35^. Samples were centrifuged at 1200 *g* for 10 minutes at 20°C. After centrifugation, serum was pipetted to a 15 mL conical tube. Serum was then aliquoted into cryovials and stored at −80°C.

### Definition of Acute Kidney Injury

We defined AKI (stage 2 or 3) as per Kidney Disease Improving Global Outcomes (KDIGO) criteria: an increase in serum creatinine of at least 2.0 times the baseline creatinine^36^. For patients with previous serum creatinine measurement available in the 365 days prior to admission, the minimum value in this period was considered the baseline creatinine. For patients without a baseline creatinine in this period, a baseline value was calculated based on an estimated glomerular filtration rate (eGFR) of 75 ml/min per 1.73 m^2^ as per the KDIGO AKI guidelines.

### Clinical data collection

We collected demographic and laboratory data collected as part of standard medical care from an institutional electronic health record (EHR) database. We defined clinical comorbidities using diagnostic codes recorded in the EHR before the current hospital admission. To account for disease severity at the time of specimen collection, we defined supplemental oxygen requirement as 0 if the patient was not receiving supplemental oxygenation or on nasal cannula, 1 if the patient was receiving non-invasive mechanical ventilation (CPAP, BIPAP), or 2 if the person was receiving invasive mechanical ventilation.

### Somalogic proteomic assay

We used the *SomaScan* discovery platform to quantify levels of protein expression^37^. The *SomaScan* platform is a highly multiplexed aptamer based proteomic assay based on Slow Off-rate Modified single-stranded DNA Aptamers (SOMAmers) capable of simultaneously detecting 4497 proteins in biological samples in the form of relative fluorescent units (RFUs). The assay was run using the standard 12 hybridization normalization control sequences to assess for variability in the Agilent plate quantification process, five human calibrator control pooled replicates, and 3 quality control pooled replicates to control for batch effects. Standard preprocessing protocols were applied as per Somalogic’s guidelines published previously^37^ The specificity and stability of the SOMAScan assay has been described previously^38^ . Briefly, the data was first normalized using the 12 hybridization controls to remove hybridization variation within a run. Then, median signal normalization is performed with calibrator samples across plates to remove variation in sample-to-sample differences attributable to variations due to pipetting, reagent concentrations, assay timings and other technical aspects. Data was then calibrated to remove assay differences between runs. Standard Somalogic acceptance criteria for quality control metrics were used (plate scale factor between 0.4 and 2.5 and 85% of QC ratios between 0.8 and 1.2). Samples with intrinsic issues such as reddish appearance or low sample volume were also removed as part of the Somalogic quality control protocol. After quality control and normalization procedures, the resulting relative fluorescence unit (RFU) values were log2 transformed.

### Dimensionality reduction

Principal component analysis (PCA) was performed using log2 transformed RFU values of all proteins. Pairwise plots of the top three principal components were plotted.

### Differential expression analysis for prevalent AKI

Using data from the AKI cohort, log_2_ transformed normalized protein values were modelled using multivariable linear regression in the Limma framework^39^ Models were adjusted for age, sex, history of chronic kidney disease (CKD), and supplemental oxygen requirement (0,1, or 2 [see above]) at the time of specimen collection. P-values were adjusted using the Benjamin-Hochberg procedure to control the false discovery rate (FDR) at 5%.

### Proteomic characterization of long-term kidney function in discovery cohort

Outpatient creatinine values measured after discharge were used to compute estimated glomerular filtration rate (eGFR) values the CKD-EPI equation. All values were taken from the EHR as part of routine clinical care with follow-up until 12/2/2021. To determine whether AKI associated protein expression correlated with post-discharge kidney function, we fit a mixed effects linear regression model with random intercept. Using the discovery cohort, protein expression of AKI-associated proteins measured at the last available timepoint during admission was used. The dependent variable was eGFR and the model was adjusted for age, sex, baseline creatinine, history of CKD, maximum AKI stage during the hospital admission, and day of eGFR measurement after hospital discharge. Models included a random effect of patient ID to adjust for correlation between eGFR values taken from the same individuals. Significance was evaluated using a t-test with Satterthwaite degrees of freedom implemented in the *ImerTest* R package^40^. P values were adjusted using the Benjamin-Hochberg procedure to control the false discovery rate (FDR) at 5%.

We then plotted the post-discharge eGFR values over time for individuals separated by protein expression tertiles (bottom 33rd percentile, middle 33rd percentile, and top 33rd percentile). We transformed data using the LOESS smoothing function as implemented in the *ggplot* R package.

### Data analysis and visualization

We performed all statistical analysis using R version 4.0.3. Protein-protein interaction (PPI) network was constructed using the Network X package in Python v3.4.10 to display a Minimum Spanning Tree (MST) using Prim’s algorithm. Network clustering was conducted using the MCL cluster algorithm and functional enrichment was carried out using the STRING^41^ database in Cytoscape^42^. Using results from a recent publication^30^, we also annotated protein quantitative trait loci (pQTLs) for the set of COVID AKI-associated proteins. For each AKI-associated protein, we determined whether cis and trans pQTL associations had been reported.

## Discussion

Using proteomic profiling in two large groups of patients hospitalized with COVID-19, we report several observations. First, we identified specific protein markers of AKI and post-discharge kidney dysfunction, both well-documented sequelae of COVID-19^4,43^. Second, in the acute phase, tubular injury and hemodynamic perturbation may play a role. Thus, characterization of the peripheral blood suggests specific large-scale perturbations of the proteome that accompany both AKI and long-term eGFR decline with implications for more specific prognostic models and targeted therapeutic development.

Based on our results, we hypothesize that COVID AKI may involve several mechanisms: tubular injury, neutrophil activation, and hemodynamic perturbation. First, we found significantly higher plasma abundances of *NGAL* (*LCN2*), a canonical marker of tubular injury that is also involved in neutrophil activation. *NGAL* is secreted by circulating neutrophils and kidney tubular epithelium in response to systemic inflammation or ischemia. Since renal tubular epithelial cells express the *angiotensin-converting enzyme 2 (ACE2)* receptor which enables SARS-CoV2 viral entry into cells, direct tubular infection may cause the release of *NGAL* into the serum and urine. This potential mechanism is supported by our results and remains a testable hypothesis. Although *NGAL* is a known marker for intrinsic AKI accompanied by tubular injury, it is relatively insensitive to pre-renal AKI caused by hemodynamic disturbance^44,45^. However, our results demonstrate higher plasma abundance of *BNP*, a protein released in the setting of volume overload as well as several cardiac structural proteins (cardiac troponin T, titin, myosin light chain 1, and sarcalumenin). This proteomic signature may represent either myocardial injury leading to decreased renal perfusion or impaired filtration by the kidney in response to injury. Myocardial injury has been previously reported in patients hospitalized with COVID-19^6^ and thus may contribute to the multifactorial nature of COVID-AKI. It is worth nothing that in addition to myocardial injury, *BNP* may also be increased in critical illness due to pro-inflammatory cytokine release.

Since COVID-AKI increases the risk of long-term eGFR decline^43^, we then sought to determine whether these two phenomena shared common proteomic markers. Surprisingly, we found that although almost half of the AKI-associated proteins were also significantly associated with post-discharge eGFR decline, the strengths of associations were not correlated. While COVID-AKI is likely caused by a combination of intrinsic tubular injury and hemodynamic disturbance in the setting of critical illness, long term eGFR decline was associated with increased expression of *trefoil factor 3 (TFF3)*, a known prognostic marker for incident CKD^46^. Trefoil factors are a class of small peptides expressed in colonic and urinary tract epithelia that play essential roles in regeneration and repair of epithelial tissue^47,48^.

Immunohistochemistry reveals *TFF3* expression is localized to the tubular epithelial cells in kidney specimens from patients with CKD^46^, suggesting that long term eGFR decline may be associated with renal tubular epithelial damage. The exact pathological role of *TFF3* in the renal tubules is unclear but it has been hypothesized to play a role in repair of kidney damage^49^. Additionally, *TFF3* release from the renal interstitium has also been hypothesized to direct the epithelial-to-mesenchymal transition (EMT) in renal interstitial fibrosis, a main pathway that leads to ESKD^46^. Our results implicate tubular damage in both AKI and long term eGFR decline suggesting that SARS-CoV2 may preferentially target this region of the nephron. While AKI in the acute setting may be a result of ischemia and decreased renal perfusion associated with critical illness, the specific elevation of *TFF3* associated with eGFR decline implicates a more general pattern of tubular injury that underlies COVID mediated kidney dysfunction. Since the *ACE2* is preferentially expressed in the tubular epithelial cells of the kidney^50,51^, the elevation of markers of tubular damage in the plasma may represent direct viral invasion of tubular epithelia cells. However, again this would need to be tested using biopsy/autopsy specimens or other mechanistic studies. Direct viral entry into the kidney remains controversial and using our current data we are not able to comment on this mechanism.

Our study should be interpreted in the context of certain limitations. First, samples were collected during the hospital course of patients with confirmed COVID-19. However, the timepoints were not systematic due to logistical challenges during the peak of the COVID-19 pandemic and thus are not standardized between patients. Since a subset of patients had AKI at the time of admission, these patients were excluded from our analysis since specimens were collected after admission. Additionally, we did not include patients who developed AKI without COVID and were unable to determine whether COVID-AKI has unique proteomic markers compared to other forms of sepsis-AKI. Thus, our AKI cases may be biased towards less severe presentations. Second, since kidney injury is usually not an isolated phenomenon in critically ill patients, the protein expression changes observed may have been partially due to damage to other organs, such as the lung, liver, and heart. However, we accounted for non-kidney damage by adjusting for the highest level of ventilatory support and thus our results are likely a reflection of kidney injury. Specifically, our results do show the importance of crosstalk between the cardiac system and the kidneys. In addition, we did not include proteomic measurements from urine specimens and thus it is unclear whether poor filtration or resorption of proteins plays a role in peripheral blood protein concentrations. For example, poor resorption of cystatin-C in the setting of AKI may have led to the increased peripheral blood cystatin-C that we report. Our study was adequately powered to detect effect sizes of greater than or equal to 1.6. Also, since we enrolled patients only from March-October 2020, we cannot generalize our findings to other COVID-19 variants and time periods. Although we adjusted our regression models for history of CKD, it is possible that unmeasured confounding due to preexisting impaired kidney function has not completely been controlled our in our analyses. Another big limitation of our study is the inability to significantly distinguish between effects of impaired filtration and true pathogenic differences in protein levels without having to conduct additional validation tests.

We were not able to exclude individuals who were lost to follow-up or died because the data was extracted from an institutional EHR. Some patients accessed care at other hospitals after discharge. This remains a limitation as well. Finally, our cohort did not include autopsy or kidney biopsy specimens. Histopathological analysis of kidney specimens is necessary to determine the mechanism of AKI and whether viral particles are present in the kidney.

In conclusion, we provide, to the best of our knowledge, the first comprehensive characterization of the plasma proteome of AKI and long term eGFR decline in hospitalized COVID-19 patients. Our results suggest in the setting of COVID-AKI and post-discharge kidney dysfunction there is evidence of tubular damage in the peripheral blood but that in the acute setting, several factors including hemodynamic disturbance and myocardial injury also play a role.

## Figures and Tables

**Figure 2:**
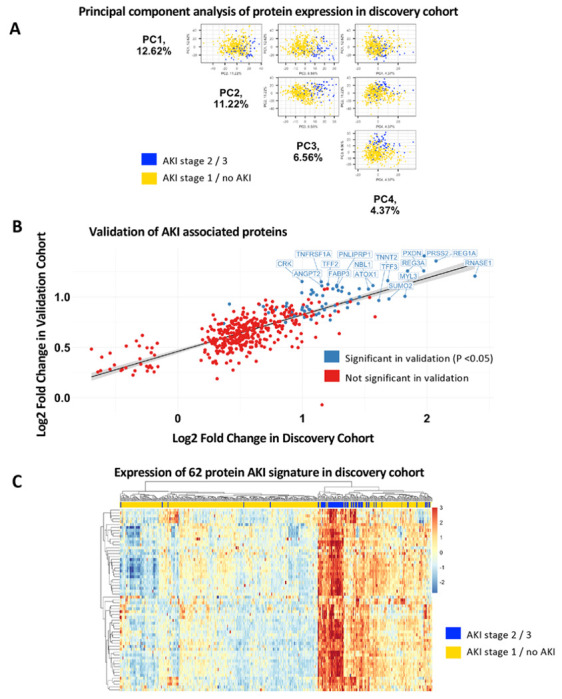
Analysis of the clinical and proteomic data. **a.** Top 3 Principal Components show separation of the sample by AKI (stage 2 or 3) case status. **b.** External validation of AKI associated proteins in the discovery cohort shows high correlation with increased risk of AKI with significance of p<0.05. (n=62) **c.** Expression heatmap shows a distinct separation of the cases and controls using the 62 significant proteins identified from the validation cohort in the discovery cohort.

**Figure 3:**
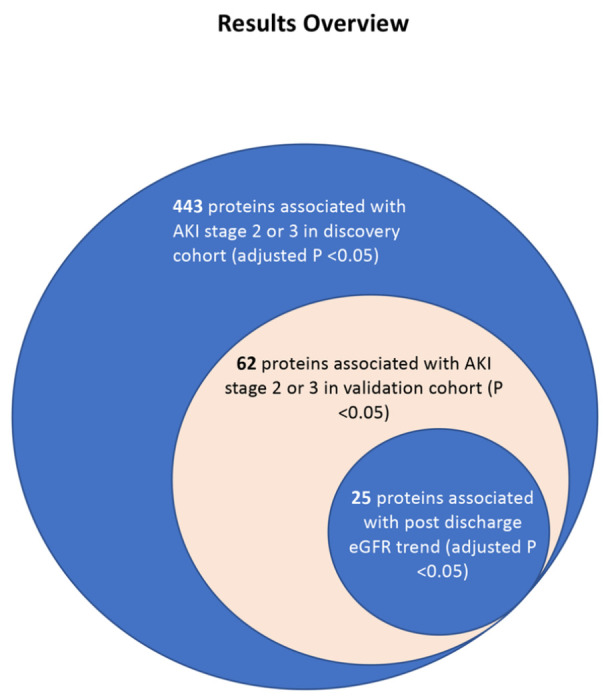
Nested Venn diagram of the analyses performed.

**Figure 4:**
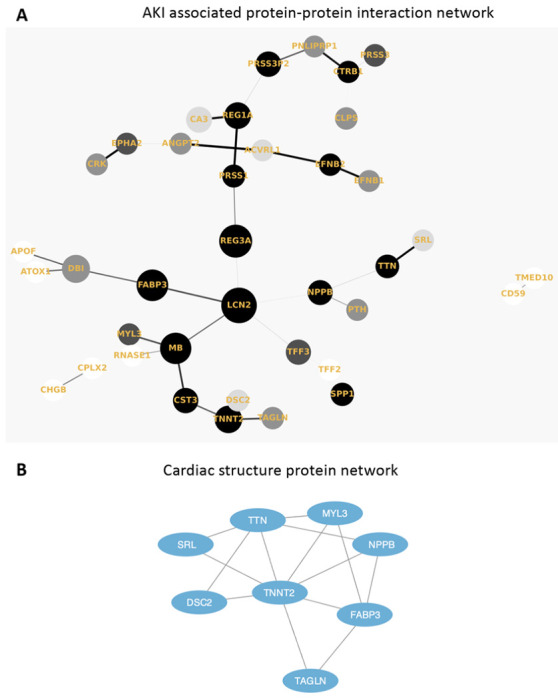
Protein-protein interaction (PPI) and clustering analysis for functional annotation of the 62 differentially expressed proteins. **a.** Protein–protein interaction (PPI) network (Minimum Spanning Tree) of the 62 overlapping AKI associated proteins with a score >0.4. The size of each node corresponds to number of interactions and the thickness of the edges represent the weight of the interactions between the nodes. **b.** MCL algorithm was used to identify tightly connected cluster of proteins which was functionally enriched for cardiac structure proteins using the STRING database.

**Figure 5:**
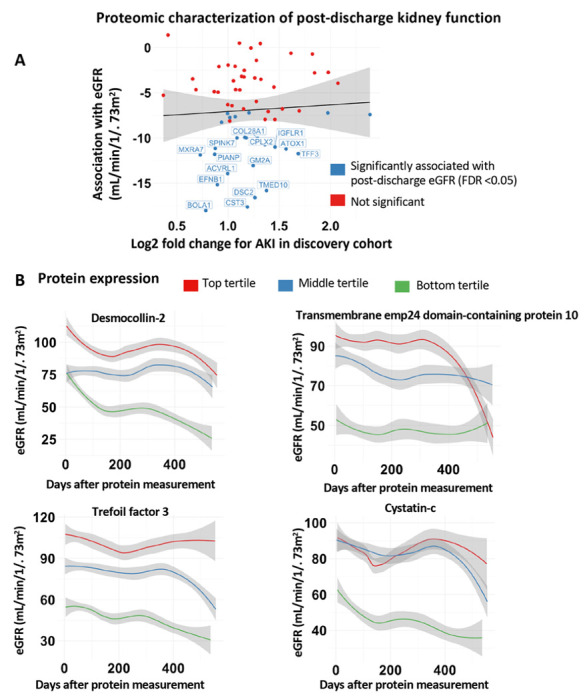
Proteomic characterization of long-term eGFR decline. **a.** Comparison of strengths of association with AKI and long term eGFR for proteins associated with AKI in both the discovery and validation cohorts (n=62). **b.** Trend in eGFR values separated by protein expression for tertiles for proteins most significantly (by P value) associated with eGFR trend.

## Data Availability

The Data is available in Synapse syn35874390 and can also be accessed by contacting the senior author, Girish Nadkarni (girish.nadkarni@mountsinai.org). Code is available at our GitHub repository Nadkarni-Lab: aki_covid_proteomics.
